# Predicting the need of aortic valve surgery in patients with chronic aortic regurgitation: a comparison between cardiovascular magnetic resonance imaging and transthoracic echocardiography

**DOI:** 10.1007/s10554-021-02255-7

**Published:** 2021-05-18

**Authors:** M. Faber, C. Sonne, S. Rosner, H. Persch, W. Reinhard, E. Hendrich, A. Will, S. Martinoff, M. Hadamitzky

**Affiliations:** 1 Department of Radiology and Nuclear Medicine, German Heart Center Munich, Hospital at Technical University Munich, Lazarettstr. 36, 80636 Munich, Germany; 2 Department of Cardiovascular Diseases, German Heart Center Munich, Hospital at Technical University Munich, Lazarettstr. 36, 80636 Munich, Germany; 3 Division of Sports and Rehabilitation Medicine, Center of Internal Medicine, University of Ulm, Leimgrubenweg 14, 89073 Ulm, Germany

**Keywords:** Aortic regurgitation, Aortic valve surgery, Prognosis, Magnetic resonance tomography, Transthoracic echocardiography

## Abstract

To compare the ability of cardiac magnetic resonance tomography (CMR) and transthoracic echocardiography (TTE) to predict the need for valve surgery in patients with chronic aortic regurgitation on a mid-term basis. 66 individuals underwent assessment of aortic regurgitation (AR) both in CMR and TTE between August 2012 and April 2017. The follow-up rate was 76% with a median of 5.1 years. Cox proportional hazards method was used to assess the association of the time-to-aortic-valve-surgery, including valve replacement and reconstruction, and imaging parameters. A direct comparison of most predictive CMR and echocardiographic parameters was performed by using nested-factor-models. Sixteen patients (32%) were treated with aortic valve surgery during follow-up. Aortic valve insufficiency parameters, both of echocardiography and CMR, showed good discriminative and predictive power regarding the need of valve surgery. Within all examined parameters AR gradation derived by CMR correlated best with outcome [χ^2^ = 27.1; HR 12.2 (95% CI: 4.56, 36.8); (p < 0.0001)]. In direct comparison of both modalities, CMR assessment provided additive prognostic power beyond echocardiographic assessment of AR but not vice versa (improvement of χ^2^ from 21.4 to 28.4; p = 0.008). Nested model analysis demonstrated an overall better correlation with outcome by using both modalities compared with using echo alone with the best improvement in the moderate to severe AR range with an echo grade II out of III and a regurgitation fraction of 32% in CMR. This study corroborates the capability of CMR in direct quantification of AR and its role for guiding further treatment decisions particularly in patients with moderate AR in echocardiography.

**Supplementary Information:**

The online version contains supplementary material available at 10.1007/s10554-021-02255-7.

## Introduction

Chronic aortic regurgitation (AR) results in increased left ventricular (LV) volume and causes heightened preload and afterload, which might cause LV dilatation and dysfunction, if untreated [1, 2].

Aortic valve surgery plays a pivotal role in the management of chronic AR as it might prevent heart failure and death and can significantly improve clinical outcome [3]. Aortic valve surgery is mainly considered in patients with symptomatic stages of disease or in case of LV involvement, even in asymptomatic manifestation, as considerable chronic regurgitation might be masked and patients may remain asymptomatic for a long time.

At this stage of disease prognosis might already be declined significantly [4, 5]. This emphasizes the need of an optimal timing of aortic valve surgery [3–6]. Regarding the recommendation for aortic valve surgery accurate quantification of AR severity and LV dimensions is essential. Both, the echocardiographic and the cardiac magnetic resonance (CMR) approach are recommended for assessment of AR severity by well-established references like the American College of Cardiology and the American Society of Echocardiography [3, 6].

Echocardiographic approach remains the base for the evaluation of AR as it is commonly available, can define ventricular size and function and estimate the severity of AR.

The CMR approach shows superior reproducibility for ventricular volumes and systolic measurements. Especially fast breath-hold CMR reveals excellent results for inter-study reproducibility of LV volumes,

ejection fraction and mass, which are considered to be superior to results of 2-dimensional echocardiography [2, 7]. Yet, in the absence of a reference method it is challenging to determine which of the two modalities generates more clinically relevant information and can better predict the need of valve surgery.

Harris et al. reported in a prospective study trial at a follow up time of 4.4 ± 1.5 years CMR being better than echocardiography in predicting the need for valve surgery in patients with chronic aortic and mitral regurgitation [8]. The available studies are small and only a few focus specifically on aortic valvular disease. As most comparative studies address the questions of accuracy and reproducibility and not of clinical outcome, only very little information is given on the additive clinical value of CMR approach in comparison to echocardiographic approach [9–12]. Therefore the objective of this study was to compare on a mid-term basis, defined by a follow-up period of 5 years, the ability of CMR and echocardiography to predict valve surgery in patients with chronic AR.

## Methods

### Study cohort

Eligible for analysis were 53 patients with chronic AR undergoing both CMR and echocardiography at our institution between August 2012 and April 2017. In addition a group of 13 healthy volunteers without chronic AR was included into study population.

Exclusion criteria were general contraindications for CMR, a lack of stable sinus rhythm during examination, the existence of severe other valvular or cardiac diseases or abnormalities, former cardiac surgeries and hemodynamic instability. Severe aortic disorders like a coexisting ascending aortic aneurysm or a concurrent infection were ruled out before including patients into study population.

Also pregnancy and obesity permagna, defined as BMI > 40, led to exclusion of study population.

### CMR procedure

For image acquisition a 1.5 T scanner (Siemens Avanto, Siemens Healthineers, Erlangen) and dedicated phased array cardiac coils were used. All measurements were performed both in conventional free breathing as well as in navigator based respiratory motion compensation technique. Through-plane flow sensitive gradient echo sequences at the sinutubular junction of the aortic root with retrospective gating were utilized.

Scan parameters of the free breathing protocol were as follows:

Repetition time (TR) = 29.5 ms, echo time (TE) = 2.8 ms, field of view (FOV) 320 × 240 mm.

The slice thickness was 5 mm, the in plane resolution 1.25 × 1.25 mm. Velocity encoding was set to 150 cm/s typically. Two segments were chosen typically. Three repetitions and 30 measurements per heart cycle were done.

Scan parameters of the navigator based respiratory motion compensation protocol were as follows:

Repetition time (TR) = 12.34 ms, echo time (TE) = 2.91 ms, field of view (FOV) = 320 × 240 mm.

The slice thickness was 5 mm, the in plane resolution 1.3 × 1.3 mm. Velocity encoding was set to 150 cm/s typically and increased if necessary.

One segment was chosen typically, 75 measurements per heart cycle were done.

“Argus Flow 4.02” was used for image evaluation (Software version Syngo MR D13), aortic flow values were calculated from the aortic flow curve.

Aortic regurgitation fraction (RF) was defined as total retrograde flow divided by total anterograde flow.

AR-grade was categorized by aortic regurgitation fraction: 1–19% in mild, 20–39% in moderate and > 40% in severe AR.

Left ventricular function was assessed by using contiguous stack short axis steady state free precession cine imaging (SSFP):

Repetition time (TR) = 50.76 ms, echo time (TE) = 1.2 ms, field of view (FOV) = 340 × 240 mm.

Fifteen slices with a slice thickness of 8 mm and with a slice spacing of 0 were generated.

The in plane resolution was 1.3 × 1.3 mm. 18 segments were typically chosen and 25 measurements per heart cycle were done.

Generated images have been used to assess the end-diastolic (LVEDV; ml) and end-systolic (LVESV; ml) volume of the left ventricle.

“Argus Ventricular Function 4.01” (Software version Syngo MR D13) was used to segment manually.

end-diastolic and end-systolic endocardial contours in each slice. Left ventricular ejection fraction (EF; %) was calculated as [(LVEDV − LVESV)/LVEDV].

### Doppler echocardiography

For assessment of AR following echocardiographic machines were used: Philips iE33 and EPIQ 7G, Philips Medical Systems, Andover, Mass.

The following echocardiographic measurements were investigated:

Proximal Isovelocity Surface Area (PISA; mm^2^) was measured by using continuous-wave color Doppler circumscribing the pre-valvular volume of flow velocity above 30 cm/s.

Effective Regurgitation Orifice Area of AR (AR-EROA; mm^2^) was defined as flow velocity (2 πr^2^ × aliasing velocity) divided by peak velocity (cm/s) of the regurgitation jet as recorded by continuous-wave Doppler.

Regurgitant Volume (AR-Vol; ml) was calculated as the product of EROA and Velocity–Time-Integral (VTI).

The Enddiastolic Flow Velocity (cm/s) was measured by pulsed Doppler in end-diastole just beneath the origin of the left subclavian artery using a suprasternal notch view at the peak R wave on a simultaneously recorded electrocardiogram.

AR severity was graded according to European society of cardiology [13].

Recommended (semi-) quantitative thresholds for severe AR were used according to European Society of Cardiology (ESC) guidelines: EROA ≥ 30 mm^2^, AR-volume ≥ 60 ml, AR-RF ≥ 50%, Vena contracta (VC) > 6 mm, Pressure half time (PHT) < 200 ms.

To guarantee intra- and inter-observer variability in total 3 measurements per patient were done, including two consecutive measurements by one examiner and one additional by a second blinded examiner, both with more than 10 years of echocardiographic experience.

Investigators of both the CMR and echocardiography studies were blinded concerning AR grading of the other modality.

**Fig. 1:**
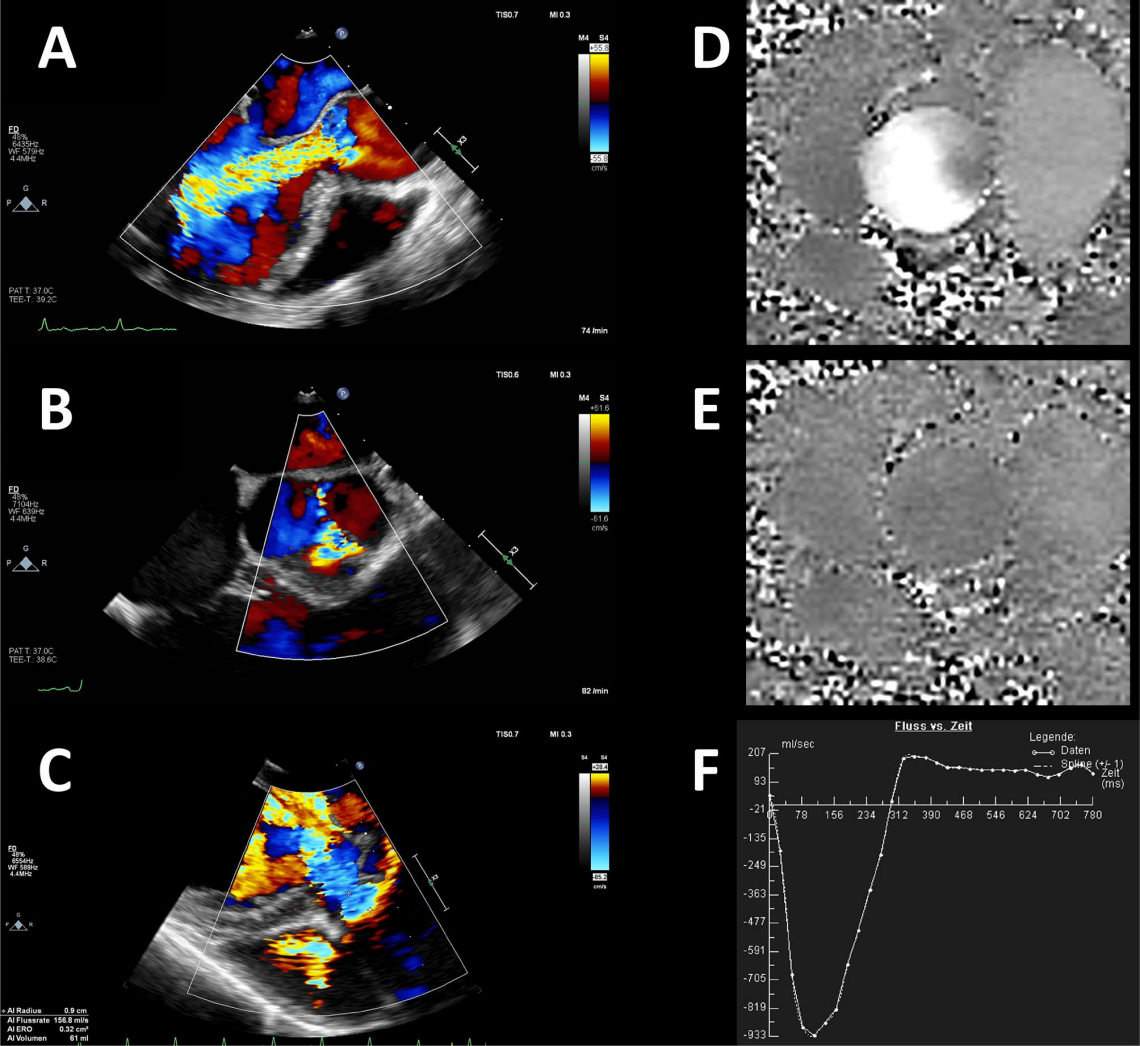
Quantification of aortic regurgitation by echo (**a**–**c**) and cardiac magnetic resonance imaging (**d**–**f**). **a**: Regurgitant jet in left ventricular outflow tract, **b**: Effective Regurgitant Orifice (short axis of aortic valve), **c**: Proximal isovelocity surface area (PISA), **d**: Phase encoded image of through plane blood velocity in systole, **e**: Same in diastole, **f**: Calculated flow-time diagram (systolic flow is depicted as negative due to patient orientation)

Figure 1 illustrates the quantification of aortic regurgitation by cardiac magnetic resonance and by echo.

### Follow-up information

Follow-up information was obtained by clinical visits or telephone contact. Verification of all stated events was ensured by hospital records or confirmation by the attending physician.

### Endpoint of the study

Aortic valve surgery, including valve replacement as well as valve reconstruction was defined as endpoint of the study. The decision to refer a patient for surgery was at the discretion of the attending physician.

### Statistical analysis

Categorical variables were described as frequencies and percentages, whereas continuous variables were characterized as means ± standard deviation or as median (IQR = interquartile range) as appropriate.

All statistical assessments rest on an event-free survival for study endpoint by using Kaplan–Meier method.

Cox proportional hazards method was used for calculating hazard ratios and carrying out multivariable analyses. Concordance c-indices were assessed from time-to-event data as described by Harrell et al. [14, 15].

Since C-statistics has limited sensitivity to compare models of small size we used an approach of reciprocal nested models for comparing CMR and echo parameters.

Therefore the best CMR parameter was combined over selected echo parameters and vice versa in order to elucidate whether one modality can improve the predictive value of the other or if both modalities are necessary to get an optimal result.

All statistical analyses were carried out two-sided and a significance level of 5% was defined.

The statistical package R version 3.6.0 including the package rms was used for statistical tests.

## Results

### Study population

The study population comprises 66 individuals undergoing assessment of aortic regurgitation (AR) both in CMR and echocardiography. Among these, 53 were patients with known or suspected chronic AR, 13 were healthy individuals without known AR. Out of 66 examined patients 50 could be included into the study as follow-up was successful (follow-up rate of 76% at a median of 5.1 years).

The mean age of the study population was 52.4 years, 35 patients were male and 15 female.

### Endpoint and clinical correlation

Overall 16 patients underwent aortic valve surgery. Among these 11 patients underwent aortic valve replacement and 5 valve reconstruction. In comparison to patients without a history of aortic valve surgery, they were significantly older [62.5 (57.4, 69.4) years vs. 46.8 (35.5, 55.3) years; p = 0.003] and showed a higher prevalence of arterial hypertension [12 (75%) vs. 12 (35.5%); p = 0.015] and stated more often a positive family history of coronary artery disease [5 (31.2%) vs. 2 (5.88%); p = 0.027].

Detailed data are summarized in Table 1.

**Table 1:** Patient characteristics

	Study cohort n = 50	No event n = 34	Event n = 16	P-value
Male gender	35 (70%)	21 (61.8%)	14 (87.55)	0.099
Age	52.4 (38.7, 62.5)	46.8 (35.5, 55.3)	62.5 (57.4, 69.4)	0.003
NYHA				0.87
NYHA 1	37 (75.5%)	26 (76.5%)	11 (73.3%)	0.97
NYHA 1.5	1 (2.04%)	1 (2.94%)	0 (0%)	0.8
NYHA 2	4 (8.16%)	3 (8.82%)	1 (6.67%)	0.97
NYHA 2.5	4 (8.16%)	2 (5.88%)	2 (13.3%)	0.68
NYHA 3	3 (6.12%)	2 (5.88%)	1 (6.67%)	0.99
CAD	5 (10%)	2 (5.88%)	3 (18.8%)	0.31
Arterial Hypertension	24 (48%)	12 (35.3%)	12 (75%)	0.015
Hypercholesterolemia	17 (34%)	9 (26.5%)	8 (50%)	0.12
Diabetes mellitus	2 (4%)	1 (2.94%)	1 (6.25%)	0.54
Smoking	5 (10%)	5 (14.7%)	0 (0%)	0.16
Family history of CAD	7 (14%)	2 (5.88%)	5 (31.2%)	0.027
ACE inhibitors	30 (60%)	17 (50%)	13 (81.2%)	0.062
Beta-blockers	22 (44%)	13 (38.2%)	9 (56.2%)	0.36
Alpha-blockers	3 (6%)	0 (0%)	3 (18.8%)	0.029
Calcium-antagonsists	9 (18%)	2 (5.88%)	7 (43.8%)	0.0027
Diuretic	17 (34%)	8 (23.5%)	9 (56.2%)	0.03
Thiazide-diuretics	16 (32%)	8 (23.5%)	8 (50%)	0.1
Loop-diuretics	2 (4%)	1 (2.94%)	1 (6.25%)	0.54
Spironolactone	2 (4%)	0 (0%)	2 (12.5%)	0.098
ASS	14 (28%)	8 (23.5%)	6 (37.5%)	0.33
Statin	14 (28%)	6 (17.6%)	8 (50%)	0.04

### CMR results

Mean LV-EF of study population was 59 ± 10.5%. Patients who underwent aortic valve surgery showed a lower mean LV-EF compared to patients without surgery (52.4 ± 15% vs. 62.9 ± 5.13%; p = 0.015). They showed a higher mean value of AR (48 ± 19.8% vs. 16.1 ± 16.2%; p < 0.0001), irrespective of performed sequences (standard sequences in conventional free breathing vs. sequences in navigator based respiratory motion compensation).

Moreover, patients with aortic valve procedure showed higher LV endsystolic volumes (151 ± 105 ml vs. 74.6 ± 25.6 ml, p < 0.0001) and higher enddiastolic volumes (260 ± 71.5 ml vs. 185 ± 55.9 ml, p = 0.00025) than patients without aortic valve procedure.

### Echocardiographic results

Patients with aortic valve surgery showed overall a higher grade of aortic insufficiency classified with the recommended echocardiographic AR parameters and greater LV dilation than patients without surgery. They showed higher LV-EDV (200 ± 74.6 ml vs. 129 ± 47.4 ml; p = 0.0032) and LV-ESV (101 ± 59.7 ml vs. 50.3 ± 22.3 ml; p = 0.006) and a higher clinical gradation of AR (2.59 ± 0.417% vs. 1.32 ± 1.08%; p < 0.0001).

Also vena contracta (6.14 ± 1.8 mm vs. 3.11 ± 2.49 mm; p < 0.0001), PISA-Radius (7.57 ± 0.938 mm vs. 4.06 ± 3.58, p < 0.0001), EROA (0.264 ± 0.0467 mm^2^ vs. 0.125 ± 0.144 mm^2^, p < 0.0001), regurgitant volume (61.6 ± 15.1 ml vs. 30.1 ± 34 ml, p < 0.0001) was higher and PHT was lower (424 ± 132 ms vs. 627 ± 235 ms, p = 0.00023) in patients with aortic valve surgery than in patients without.

Detailed CMR and echocardiographic results are given in Table 2.

**Table 2:** Univariate prognostic value of CMR and echocardiographic parameters

	No event n = 34	Event n = 16	Hazard ratio	χ^2^	P-value	C-index
CMR results						
Aortic regurgitation (%), standard sequences	16.1 ± 16.2	48 ± 19.8	12.2 (4.56, 32.8)	27.1	< 0.0001	0.840
Aortic regurgitation (%), navigator based sequences	15.8 ± 13.9	33.5 ± 16.3	6.94 (2.36, 20.4)	14.2	0.00017	0.762
ESV (ml)	74.6 ± 25.6	151 ± 105	1.64 (1.30, 2.05)	13.8	< 0.0001	0.759
EDV (ml)	185 ± 55.9	260 ± 71.5	2.69 (1.60, 4.52)	13.4	0.00025	0.751
EF (%)	62.9 ± 5.13	52.4 ± 15	0.49 (0.34, 0.70)	12	0.00054	0.728
Echocardiographic results						
Aortic regurgitation (degree)	1.32 ± 1.08	2.59 ± 0.417	16.7 (3.41, 82.3)	21.4	< 0.0001	0.816
Vena contracta (VC) (mm)	3.11 ± 2.49	6.14 ± 1.8	12.6 (2.9, 53.9)	15.3	< 0.0001	0.766
Enddiastolic flow velocity (cm/s)	44.5 ± 51.3	114 ± 28.8	6.23 (2.17, 17.8)	13.7	< 0.0001	0.825
EDV (ml)	129 ± 47.4	200 ± 74.6	3.54 (1.78, 7.05)	12.2	< 0.0001	0.752
ESV (ml)	50.3 ± 22.3	101 ± 59.7	1.88 (1.34, 2.63)	10.8	< 0.0001	0.754
Proximal isovelocity surface area (PISA) (mm)	4.06 ± 3.58	7.57 ± 0.938	6.74 (1.92, 23.7)	10.5	< 0.0001	0.786
Pressure half time (PHT) (ms)	627 ± 235	424 ± 132	0.19 (0.05, 0.66)	8.77	0.00023	0.709
Effective regurgitation orifice area of AR (EROA) (mm^2^)	0.125 ± 0.144	0.264 ± 0.0467	3.0 (1.5, 6.3)	7.47	< 0.0001	0.814
Regurgitant volume (ml)	30.1 ± 34	61.6 ± 15.1	3.1 (1.4, 6.6)	7.01	< 0.0001	0.771
Velocity–time-integral (VTI)	143 ± 144	237 ± 50.7	3.1 (0.87, 10.9)	3.39	0.066	0.639
Peak velocity (cm/s)	247 ± 243	376 ± 175	2.6 (0.77, 8.7)	2.6	0.05	0.629

### Discriminatory ability and predictive power of CMR and echocardiographic parameters

**Fig. 2:**
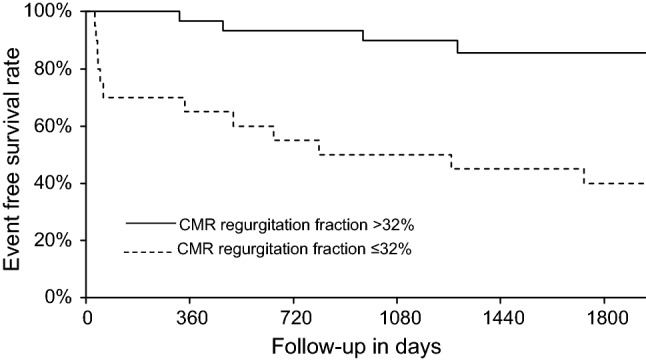
Kaplan–Meyer plot for survival free of aortic valve repair grouped by CMR aortic regurgitation fraction (plotted with R rms package)

The parameter AR gradation derived by CMR correlated best with outcome within all selected CMR and echocardiographic parameters with the highest univariate χ^2^ of 27.1 and a significantly elevated hazard ratio of 12.2 (95% CI: 4.56, 36.8); (p < 0.0001). A Kaplan–Meyer plot for a cutoff of 32% regurgitation fraction is shown in Fig. 2.

Within all selected CMR parameters AR gradation showed the best result, irrespective of performed sequences (standard sequences in conventional free breathing vs. sequences in navigator based respiratory motion compensation). Second and third best result was shown for LV-ESV and LV-EDV with a χ^2^ of 13.8 and 13.4, respectively.

Within selected echocardiographic parameters the integrated gradation of AR correlated best with endpoint with an univariate χ^2^ of 21.4 and a hazard ratio of 16.7 (95% CI: 3.41, 82.3; p < 0.001). Other echocardiographic parameters which were able to predict endpoint were vena contracta and PISA with a χ^2^ of 15.3 and a HR of 12.6 (95% CI: 2.92, 53.9; p < 0.001) and a χ^2^ of 10.5 and a HR of 6.74 (95% CI: 1.92, 23.7); p = 0.012, respectively.

### Comparison of the different parameters in a nested-factor-model

In direct comparison between AR gradation assessed by CMR and echo, the combined multivariate model (χ^2^ = 28.4) was significantly better than AR gradation in echo alone (χ^2^ = 21.4; p = 0.008), whereas it was not superior to AR gradation in CMR alone (χ^2^ = 27.1; p = 0.24), see also Table 3.

**Table 3:** Nested factor model for CMR and echocardiographic assessment of aortic regurgitation

	χ^2^ univariate	χ^2^ multivariate	P for difference
Combined vs. echo aortic regurgitation	21.4	28.4	0.0079
Combined vs. CMR aortic regurgitation	27.1	28.4	0.24

AR gradation in CMR and echo improve prediction compared to any other single parameter both in CMR and echo. For example AR quantification significantly improves prediction of end diastolic volume with a χ^2^ for improvement of 14.2 (p = 0.00016) and 9.61 (p = 0.0019) for CMR and echo respectively.

Detailed information is given in Table 4.

**Table 4:** Improvement of prediction by adding CMR AR-quantification and echo integrative grading to selected single CMR- and Echoparameters

	+ CMR AR	+ ECHO AR
CMR EDV	14.2 (p = 0.00016)	12.2 (p = 0.00047)
CMR ESV	15.0 (p = 0.00011)	13.5 (p = 0.00029)
CMR EF	15.4 (p < 0.0001)	16.1 (p < 0.0001)
Echo V. contracta	10.1 (p = 0.0015)	7.06 (p = 0.0079)
Echo enddiastolic flow rate	11.5 (p = 0.00070)	6.39 (p = 0.011)
Echo PISA	12.3 (p = 0.00045)	8.98 (p = 0.0027)
Echo EDV	10.3 (p = 0.0013)	9.61 (p = 0.0019)
Echo ESV	11.5 (p = 0.00070)	12.5 (p = 0.00039)

Likelyhood χ^2^ and p-value for comparison between nested model and single value

**Fig. 3:**
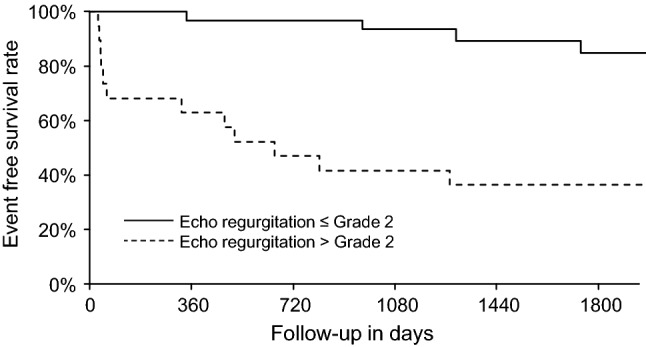
Kaplan–Meyer plot for survival free of aortic valve repair grouped by Echo aortic regurgitation gradation (plotted with R rms package)

Figure 3 shows the Kaplan–Meyer plot for survival free of aortic valve repair grouped by Echo aortic regurgitation gradation.

## Discussion

The optimal timing of aortic valve surgery remains controversial [3–6]. Accurate quantification of AR severity and LV dimensions are essential parameters for this decision [3, 6]. Even though echocardiography and CMR are recommended for assessment of AR severity [3, 6], it remains challenging to determine which of the two modalities generates more clinically relevant information and can better predict the need of valve surgery. The results of this mid-term follow up study provide several important insights into multimodality AR assessment and the prediction of valve surgery in patients with chronic AR:

1. Aortic valve insufficiency parameters, both of echocardiography and CMR, showed good discriminative and predictive power regarding valve surgery.
2. There was a good correlation of aortic insufficiency gradation between echocardiography and CMR.
3. In direct comparison of both modalities, CMR assessment of AR provides additive prognostic power beyond echocardiographic assessment of AR but not vice versa.

Both the integrative AR gradation of an experienced reader in echocardiography and the direct AR quantification in CMR show an excellent prediction of valve surgery.

Of the different AR parameters in echocardiography, vena contracta correlated best with outcome, but remained inferior to the integrative gradation recommended by the ESC guidelines. Similar results were found by Messika-Zeitoun et al., although this study did not use a clinical endpoint [16]. In both modalities, LV-diameters had only moderate correlation with outcome. Similarly, Myerson et al. observed in a multicenter study, based on data of 113 patients with at least moderate chronic AR, at a mean follow-up period of 2.6 ± 2.1 years a higher discriminatory ability for aortic valve surgery of AR fraction than of LVEDV and concluded that LV-dilatation develops only late in the course of AR deterioration [17]. Similar results were found for echocardiography by Tarasoutchi et al. [18].

An innovative work in progress sequence using free breathing navigator and thus reducing motion artifacts and repetitive image acquisition and allowing for increased temporal resolution did not improve correlation with the endpoint. Obviously the robustness of averaging information from three repetitions in the standard sequence outperforms the information gained from the higher resolution and the somehow higher variability of the work in progress sequence.

Initial evaluation of AR is almost always done by echo. In most cases the results are clear cut, but the limitations particularly in patients with suboptimal echocardiography conditions or inconclusive Doppler findings are well known. To address this clinical situation we used a nested model and could demonstrate an overall better correlation with outcome by using both modalities compared with echo alone. As can be seen in Fig. 2 this improvement is most pronounced in the moderate to severe AR range with an echo grade II out of III and a regurgitation fraction of 32% in CMR. This finding goes in line with other studies.

Harris et al. reported in a prospective study trial at a follow up time of 4.4 ± 1.5 years CMR being better than echocardiography in predicting the need for valve surgery in patients with chronic aortic and mitral regurgitation. Primary endpoint was defined as a combination of heart failure hospitalization and the need for valve surgery.

In comparison of both modalities, AR-volume assessed by CMR was more predictive for outcome than assessed by echocardiography with an AUC being significantly greater for CMR-derived regurgitant volume (0.9 vs. 0.67; p < 0.005) [8]. Neisius et al. showed stronger correlation of post aortic valve replacement LV remodeling with CMR AR grade than with echocardiography in 24 patients undergoing valve repair for chronic AR [10].

## Conclusion

The results of this study corroborate the capability of CMR in direct quantification of aortic regurgitation.

While the slightly better outcome correlation does not justify the higher expenditure in all cases, CMR can play an important role for guiding further treatment particularly in patients with moderate aortic regurgitation or inconclusive findings in echocardiography.

## Limitations

This is a single-center observational study. Results could be influenced by geographical patient characteristics as well as by the local investigation algorithm. In addition, the decision for or against surgery is always based on clinical judgment and may not only based on disease severity but also influenced by other factors.

The small number of patients did not allow for further subgroup analyses.

Due to the long recruitment and follow-up duration image acquisition techniques both for CMR and Echo do not represent the current state of the art but clinical routine in the early second decade of the century. Results might be different with current state of the art examination techniques.

## Supplementary Information

Below is the link to the electronic supplementary material.

Supplementary file1 (MP4 755 kb) QUANTIFICATION OF AORTIC REGURGITATION BY CARDIAC MAGNETIC RESONANCE IMAGING: Phase encoded image of through plane blood velocity in systole

Supplementary file2 (AVI 2055 kb) QUANTIFICATION OF AORTIC REGURGITATION BY ECHO: Regurgitant jet in left ventricular outflow tract

## Data Availability

The data supporting the findings of this study are available from the corresponding author, upon reasonable request.

## Abbreviations

AR: Aortic regurgitation
BMI: Body mass index
CAD: Coronary artery disease
HR: Hazard ratio
IQR: Interquartile range
LV: Left ventricle
CMR: Cardiac magnetic resonance tomography
TTE: Transthoracic echocardiography

## References

[1] Starling MR, Kirsh MM, Montgomery DG, Gross MD (1991). Mechanisms for left ventricular systolic dysfunction in aortic regurgitation: importance for predicting the functional response to aortic valve replacement. J Am Coll Cardiol.

[2] Lee JC, Branch KR, Hamilton-Craig C, Krieger EV (2018). Evaluation of aortic regurgitation with cardiac magnetic resonance imaging: a systematic review. Heart.

[3] Nishimura RA, Otto CM, Bonow RO, Carabello BA, Erwin JP, Guyton RA, O'Gara PT, Ruiz CE, Skubas NJ, Sorajja P, Sundt TM, Thomas JD (2014). 2014 AHA/ACC guideline for the management of patients with valvular heart disease: executive summary: a report of the American College of Cardiology/American Heart Association Task Force on Practice Guidelines. Circulation.

[4] Turina J, Milincic J, Seifert B, Turina M (1998). Valve replacement in chronic aortic regurgitation. True predictors of survival after extended follow-up. Circulation.

[5] Chaliki HP, Mohty D, Avierinos JF, Scott CG, Schaff HV, Tajik AJ, Enriquez-Sarano M (2002). Outcomes after aortic valve replacement in patients with severe aortic regurgitation and markedly reduced left ventricular function. Circulation.

[6] Zoghbi WA, Adams D, Bonow RO, Enriquez-Sarano M, Foster E, Grayburn PA, Hahn RT, Han Y, Hung J, Lang RM, Little SH, Shah DJ, Shernan S, Thavendiranathan P, Thomas JD, Weissman NJ (2017). Recommendations for Noninvasive Evaluation of Native Valvular Regurgitation: A Report from the American Society of Echocardiography Developed in Collaboration with the Society for Cardiovascular Magnetic Resonance. J Am Soc Echocardiogr.

[7] Grothues F, Smith GC, Moon JC, Bellenger NG, Collins P, Klein HU, Pennell DJ (2002). Comparison of interstudy reproducibility of cardiovascular magnetic resonance with two-dimensional echocardiography in normal subjects and in patients with heart failure or left ventricular hypertrophy. Am J Cardiol.

[8] Harris AW, Krieger EV, Kim M, Cawley PJ, Owens DS, Hamilton-Craig C, Maki J, Otto CM (2017). Cardiac Magnetic Resonance Imaging Versus Transthoracic Echocardiography for Prediction of Outcomes in Chronic Aortic or Mitral Regurgitation. Am J Cardiol.

[9] Cawley PJ, Hamilton-Craig C, Owens DS, Krieger EV, Strugnell WE, Mitsumori L, D'Jang CL, Schwaegler RG, Nguyen KQ, Nguyen B, Maki JH, Otto CM (2013). Prospective comparison of valve regurgitation quantitation by cardiac magnetic resonance imaging and transthoracic echocardiography. Circ Cardiovasc Imaging.

[10] Neisius U, Tsao CW, Hauser TH, Patel AD, Pierce P, Ben-Assa E, Nezafat R, Manning WJ (2020). Aortic regurgitation assessment by cardiovascular magnetic resonance imaging and transthoracic echocardiography: intermodality disagreement impacting on prediction of post-surgical left ventricular remodeling. Int J Cardiovasc Imaging.

[11] Gabriel RS, Renapurkar R, Bolen MA, Verhaert D, Leiber M, Flamm SD, Griffin BP, Desai MY (2011). Comparison of severity of aortic regurgitation by cardiovascular magnetic resonance versus transthoracic echocardiography. Am J Cardiol.

[12] Kutty S, Whitehead KK, Natarajan S, Harris MA, Wernovsky G, Fogel MA (2009). Qualitative echocardiographic assessment of aortic valve regurgitation with quantitative cardiac magnetic resonance: a comparative study. Pediatr Cardiol.

[13] Lancellotti P, Moura L, Pierard LA, Agricola E, Popescu BA, Tribouilloy C, Hagendorff A, Monin JL, Badano L, Zamorano JL (2010). European Association of Echocardiography recommendations for the assessment of valvular regurgitation. Part 2: mitral and tricuspid regurgitation (native valve disease). Eur J Echocardiogr.

[14] Harrell FE, Lee KL, Mark DB (1996). Multivariable prognostic models: issues in developing models, evaluating assumptions and adequacy, and measuring and reducing errors. Stat Med.

[15] Harrell FE (2017). rms: regression modeling strategies. Springer series in statistics.

[16] Messika-Zeitoun D, Detaint D, Leye M, Tribouilloy C, Michelena HI, Pislaru S, Brochet E, Iung B, Vahanian A, Enriquez-Sarano M (2011). Comparison of semiquantitative and quantitative assessment of severity of aortic regurgitation: clinical implications. J Am Soc Echocardiogr.

[17] Myerson SG, d'Arcy J, Mohiaddin R, Greenwood JP, Karamitsos TD, Francis JM, Banning AP, Christiansen JP, Neubauer S (2012). Aortic regurgitation quantification using cardiovascular magnetic resonance: association with clinical outcome. Circulation.

[18] Tarasoutchi F, Grinberg M, Filho JP, Izaki M, Cardoso LF, Pomerantezeff P, Nuschbacher A, da Luz PL (1999). Symptoms, left ventricular function, and timing of valve replacement surgery in patients with aortic regurgitation. Am Heart J.

